# New insights into Rett syndrome pathogenesis: defining the role of MEPC2 in DNA damage

**DOI:** 10.3389/fneur.2026.1711771

**Published:** 2026-05-11

**Authors:** Niloufar Mansooralavi, William E. Lowry

**Affiliations:** 1 Molecular Biology Institute, IDDRC, UCLA, Los Angeles, CA, United States; 2 Department of Molecular Cell and Developmental Biology, IDDRC, UCLA, Los Angeles, CA, United States; 3 Broad Center for Regenerative Medicine, IDDRC, UCLA, Los Angeles, CA, Jonsson Comprehensive Cancer Center, IDDRC, UCLA, Los Angeles, CA, United States; 4 Division of Dermatology, DGSOM, IDDRC, UCLA, Los Angeles, CA, United States

**Keywords:** chromatinopathy, DNA damage, human neuron, MECP2, Rett syndrome, DNA repair, senescence

## Abstract

Rett Syndrome (RTT), a severe neurodevelopmental disorder, is caused by mutations in the X-linked MECP2 gene, which encodes a key chromatin-modifying protein. Originally described as a transcriptional repressor due to its ability to bind methylated DNA, RTT pathology was first to the misregulation of target genes. However, we present a synthesis of recent research that could re-frame the etiology of the disease. This model proposes that MECP2 deficiency triggers a cellular stress response that is one of the direct and primary causes of RTT pathology. The central hypothesis emerging from this body of work is that the loss of MECP2 function directly impairs cellular machinery for DNA repair. This failure in genomic maintenance results in an accumulation of DNA damage, which acts as a primary trigger for a senescence response triggered by the p53 pathway. This senescent state initiates a cascade of downstream physiological deficits, including severe metabolic dysfunction, reduced dendritic branching, and impaired synaptic activity. This model represents a repositioning of RTT from a disorder of simple transcriptional misregulation to a complex pathology rooted in a fundamental failure of genomic integrity and cellular homeostasis. The findings open new, targeted therapeutic avenues and offer not only a potential mechanistic understanding of RTT, and potentially other Intellectual Disability syndromes caused by mutations in genes that act in the same pathways.

## Background

Methyl-CpG binding protein 2 (MECP2) is a protein encoded by a gene on the X chromosome, the disruption of which is the primary cause of Rett Syndrome (RTT) (1, 2). MECP2’s canonical function has been widely understood as that of a transcriptional repressor (2, 3). It is known to bind to methylated DNA, particularly at methylated CpG dinucleotides, and facilitates gene silencing by recruiting chromatin-remodeling complexes and co-repressors, such as histone deacetylases (2, 4–6). This function positions MECP2 as a critical epigenetic regulator involved in maintaining chromatin structure and controlling gene expression.

While MECP2 is found in most tissues and cell types, the expression is highest in the brain, particularly in mature neurons of the central nervous system (7, 8). The level of MECP2 protein correlates with neural maturation and synapse formation, suggesting a vital role in the later stages of neuronal development and maintenance. Accordingly, RTT is a severe neurodevelopmental disorder, affecting mostly female heterozygotes, as males with the mutation usually do not survive. The syndrome is characterized by a postnatal loss of neurophysiological function, cognitive decline, intellectual disability, and microcephaly from 6 to 18 months of age (9–11).

Despite decades of research, the precise mechanism by which MECP2 mutations lead to RTT symptoms has remained unclear. These large number of studies has led to at least one new treatment, Trofinetide (12, 13), which treats symptoms but does not reverse the course of the disease. Several groups are now attempting to restore expression of wildtype MECP2 in Rett neurons by reactivating the MECP2 locus following X-Chromosome Inactivation (XCI), or by delivering a wildtype copy via gene therapy (14, 15). While these clinical efforts continue, many are still grappling with understanding the etiology of the disease by delineating the molecular role of MECP2 in neurons. A large number of studies have attempted to identify a consistent set of differentially expressed genes (DEGs) in MECP2-deficient neurons, but have often yielded conflicting results, with only a few specific gene targets, such as BDNF and IGF (related to Trofinetide), consistently identified (16–18). This lack of a clear transcriptional signature has led to a shifting perspective on MECP2’s role, suggesting it may function as a more complex chromatin modulator rather than a simple transcriptional repressor (9, 16, 19). This evolving understanding highlights the need to explore higher-order molecular and cellular pathologies that may be responsible for the broad and profound physiological defects observed in RTT.

## Studies to understand the consequence of loss of MECP2 in neurons

The pervasive yet subtle transcriptional changes observed in MECP2-deficient cells have necessitated a new approach to understanding RTT pathogenesis. Rather than focusing on individual gene targets, a more comprehensive view considers how MECP2 deficiency impacts fundamental cellular processes (20–24). The collective research suggests that RTT pathology arises not from a localized transcriptional defect, but from a systemic failure in cellular homeostasis, manifesting as pathologies typically associated with aging (21, 25, 26). This integrative approach posits that the molecular and physiological dysfunction in RTT neurons is a consequence of a broader cellular stress response (27–30). This hypothesis is supported by findings across various model systems, from human iPSC-derived neurons to mouse and rat models, which reveal a consistent link between MECP2 deficiency and pathologies like cellular senescence, DNA damage, and metabolic dysfunction.

A growing body of evidence establishes a link between MECP2 deficiency and the induction of a premature senescence program (27, 30–34). This is consistently observed across different cell types and species, suggesting it is a fundamental pathological consequence of MECP2 loss rather than a tissue-specific effect. In one of the first studies to implicate senescence, the Galderisi group showed that mesenchymal stromal cells (MSCs) derived from the bone marrow of an RTT patient showed signs of precocious senescence when compared to healthy controls (31, 32). This was demonstrated by an increased percentage of cells with senescence-associated *β*-galactosidase activity, a hallmark of this cellular state. This observation was further validated in a mouse model of RTT (Mecp2+/− mice), where MSCs exhibited a higher percentage of senescent cells and a lower degree of proliferation and apoptosis (27). Flow cytometry analysis of these cells showed a significant reduction in the S-phase population and a corresponding increase in G1-phase cells, indicating a cell cycle arrest consistent with senescence. The link between MECP2 and senescence is not limited to mesenchymal stem cells. Studies using a human neuroblastoma cell line with a partially silenced MECP2 gene showed that this impairment triggered senescence, which in turn perturbed neural cell fate and neuronal maintenance. This finding was further corroborated in a mouse model where MECP2-deficient neural stem cells were also found to be prone to premature senescence and showed reduced capacity to cope with genotoxic stress.

This senescence accumulation in post-mitotic cell populations such as neurons is an indication of enhanced aging-like phenotype. An *in vivo* study by Jurk et al. describes a senescence-like phenotype in post-mitotic neurons (35–37). In this study it was reported that senescence markers accumulated in aged but not young mice. Using single- and double-knockout models, the authors showed that this phenotype was triggered by a telomere-driven DNA damage response and p21-dependent signaling (38).

Compelling evidence also comes from *in vitro* models using human induced pluripotent stem cells (hiPSCs) derived from RTT patients (30). Neurons differentiated from these hiPSCs in the absence of MECP2 displayed clear signs of stress, including the induction of a senescence-associated secretory phenotype (SASP) program and robust *β*-galactosidase activity. The consistent observation of senescence across non-neuronal MSCs, neuronal progenitor cells, and mature neurons strongly suggests that the core dysfunction caused by MECP2 loss is not confined to a single tissue but is more universal, with neurons perhaps being particularly sensitive to the downstream consequences.

Notably, while cellular senescence and DNA damage emerge as consistent consequences of MECP2 loss, there are many plausible models of Rett pathogenesis, particularly those centered on methylation dysregulation, chromatin remodeling, and impaired neuronal plasticity. Beyond Rett syndrome, evidence from other neurodevelopmental disorders further supports the idea that impaired genome maintenance is a recurring vulnerability in conditions caused by chromatin or nucleic acid regulatory defects. Aicardi-Goutières syndrome (AGS), a monogenic neurodevelopmental disorder caused by mutations in genes involved in nucleic acid metabolism and processing (including RNASEH2A/B/C, SAMHD1, and TREX1), have been shown to exhibit elevated DNA damage, R-loop accumulation, and chronic activation of DNA damage response pathways in patient-derived cells and animal models. These studies demonstrate that defective resolution of nucleic acid structures leads to intrinsic genomic instability, which precedes and contributes to downstream neuroinflammatory and neurodevelopmental phenotypes. Similarly, chromatinopathies such as Coffin-Siris syndrome, caused by mutations in SWI/SNF (BAF) chromatin remodeling complex components, implicate pathways that regulate chromatin accessibility and are increasingly recognized as important for DNA damage recognition and repair. While transcriptional dysregulation remains a central feature of these disorders, the convergence of chromatin remodeling defects and genome instability across multiple neurodevelopmental syndromes suggests that impaired DNA repair and maintenance may represent a shared pathological axis rather than a disease-specific anomaly (39–41).

## The molecular signature of senescence in Rett

The senescence phenotype in MECP2-deficient cells is driven by the activation of well-established cell cycle regulatory pathways. In human neurons lacking MECP2, there is a clear and consistent induction of p53, which, in turn, activates various downstream target genes. This is evidenced by a significant upregulation of canonical p53 targets such as P21, GADD45, and DDB2, as detected by RT-PCR in patient-derived neurons and brain tissue. In MECP2-deficient mesenchymal stromal cells, the senescence phenotype is accompanied by an increase in the mRNA levels of the retinoblastoma family genes (Rb1 and Rb2/P130) and the cyclin-dependent kinase inhibitor (CDKI) P16INK4A (31). This activation of the p16-Rb pathway is a well-known mechanism for inducing cellular senescence and is consistent with the observed G1 cell cycle arrest and reduced proliferation in these cells. The activation of both the p53 and p16 pathways highlights a concerted cellular effort to halt proliferation and enter a senescent state in response to the underlying stress caused by MECP2 loss.

The and consistent finding of senescence across different cell types and species in the absence of functional MECP2 leads to a significant conclusion: the core pathological process is a conserved cellular stress response rather than a tissue-specific defect. While RTT primarily manifests as a neurodevelopmental disorder, the fact that non-neuronal cells like MSCs also exhibit senescence suggests that MECP2’s role extends beyond neuronal function to a more fundamental aspect of cellular maintenance. The high expression of MECP2 in mature neurons likely means these cells are uniquely vulnerable to the loss of this homeostatic regulator, which is why the disease pathology is predominantly neurological. The induction of a robust senescence program, complete with a SASP, represents a coordinated cellular attempt to manage an underlying crisis, and understanding the source of this crisis is critical for a complete picture of RTT pathogenesis.

## A critical role for senescence in Rett neuron phenotypes

The pathologies of senescence and metabolic dysfunction ultimately manifest as physiological deficits of RTT, particularly in neurons. A hallmark of the syndrome is a defect in dendritic branching, which has been observed in virtually all RTT models to date, both *in vivo* and *in vitro*. Studies on human iPSC-derived neurons show a statistically significant decrease in dendritic complexity in the absence of MECP2 (30). This structural defect is a consequence of the upstream cellular stress response. The induction of p53, a key regulator of senescence, was shown to be causally linked to this defect, as its inhibition with a small molecule, Pifithrin-*α*, was sufficient to restore dendritic complexity in MECP2-null neurons (30). Furthermore, an additional study with Rett patient derived organoids uncovered defects in neuronal networks that were restored by Pifithrin treatment (42). Together, these data suggest that some of the phenotypes that emerge in patients could be due a P53-mediated stress response as opposed to the initial molecular triggering event caused by loss of MECP2.

## Direct evidence of elevated DNA damage

The core pathological event triggering senescence and subsequent physiological decline in RTT appears to be an accumulation of DNA damage. Specifically, an increase in *γ*-H2AX foci is observed in human iPSC-derived neurons, which indicates the presence of DNA breaks. Staining for activated ATR, a protein involved in DNA break repair, is also elevated, suggesting a specific repair pathway is engaged in response to this damage. These observations are not limited to *in vitro* models; post-mortem brain samples from RTT patients and a rat model of RTT also exhibit DNA damage and senescence transcriptional signatures. Neurons, neural progenitor cells (NPCs), and human iPSCs from isogenic RTT lines all showed increased comet tail moments in the absence of MECP2, providing direct evidence of physical DNA damage in these cells. More recently, we showed that neurons isolated from patients with Rett Syndrome show elevated DNA damage as measured by COMET assay and H2AX staining (43). In addition, these same neurons showed evidence of senescence detectable by snRNA-seq, consistent with *in vitro*-derived neurons.

Furthermore, DNA repair assays demonstrate that while wild-type neurons can repair DNA damage induced by UV irradiation within 4 h, MECP2-null neurons fail to do so, with γ-H2AX and P53BP1 foci remaining elevated (44). This finding was important, as it suggests that the pathology is not merely an increase in damage, but a fundamental defect in the cell’s ability to maintain genomic integrity.

## A novel MECP2-PARP1 interaction

The link between MECP2 deficiency and impaired DNA repair is not just correlative but it appears to be rooted in a direct physical and functional interaction with key DNA repair proteins. Through immunoprecipitation followed by mass spectrometry on wild-type neuronal nuclei, a direct interaction was discovered between MECP2 and the DNA repair machinery, most notably with PARP1. This interaction was supported by reciprocal pulldown experiments with PARP1 as the bait, which also identified MECP2 as a top interactor. This finding was also confirmed by data from external studies, which identified PARP1 and other DNA repair proteins like XRCC5, DDB1, and TOP1 as MECP2 interactors (4, 45). Functionally, there is evidence that MECP2 can even regulate the activity of PARP1. PARP1 activity was consistently found to be lower in MECP2-null neurons across multiple genetic backgrounds and even in MECP2-null rat brains. This functional connection is interesting because PARP1 is a well-established mediator of DNA repair that adds poly-ADP-ribose (PAR) moieties to proteins required for the process. The consequence of this dysfunctional interaction could suggest a causal link between DNA damage and the broader RTT pathology.

The accumulating evidence strongly suggests that DNA damage or genetic instability could be a trigger for the pathological cascade in RTT, This is supported by a series of experiments that delineate a clear causal pathway. First, inducing DNA damage in wild-type neurons with Etoposide, a known DNA-damaging agent, is sufficient to phenocopy many of the RTT defects, including a diminished oxygen consumption rate (OCR) and TCA metabolism. Conversely, deliberately inducing metabolic dysfunction in wild-type neurons with a specific inhibitor did not lead to an increase in DNA damage. Together this cascade of events is described in Boxes 1, 2.

BOX 1 **The proposed causal pathway.**

The synthesis of the provided research enables the construction of a multi-layered model of RTT pathogenesis that moves beyond previous hypotheses. This model proposes a causal pathway, beginning with the loss of MECP2 function and culminating in the hallmark symptoms of the disease.

1. Loss of MECP2 Function: The initial event is the loss or dysfunction of the MECP2 protein due to mutation.^1^ This protein is not just a transcriptional repressor but also an active player in maintaining genomic stability.
2. Impaired DNA Repair and Genotoxic Stress: The dysfunctional MECP2 protein is unable to perform its role, particularly its physical and functional interaction with key DNA repair machinery proteins, such as PARP1.^1^ The resulting impairment in DNA repair leads to an accumulation of single- and double-stranded DNA breaks, causing a state of genotoxic stress within the cell.
3. Activation of p53 and Senescence: The persistent, unrepaired DNA damage acts as a signal to the cell, activating the p53 tumor suppressor pathway.^1^ This pathway, in turn, triggers a robust cellular senescence program, characterized by a G1 cell cycle arrest, the induction of cell cycle inhibitors like p16INK4A, and the expression of a senescence-associated secretory phenotype (SASP).
4. Downstream Physiological Collapse: The senescent state and its associated pathways leads to functional deficits. The metabolic dysfunction, including impaired mitochondrial energy production and a diminished TCA cycle, is a direct consequence of senescence.^1^ Concurrently, this state compromises the high-energy demands of neuronal maintenance, leading to defects in cellular structure and communication, such as reduced dendritic branching and impaired synaptic plasticity.

BOX 2 **Novel therapeutic strategies based on the new model.**

This model of RTT pathogenesis, which identifies DNA damage as a primary trigger, provides a clear and actionable framework for developing novel therapeutic strategies.

1. Targeting p53: The research demonstrates that p53 inhibition can rescue dendritic branching defects in MECP2-null neurons. This suggests that targeting the p53 pathway with small molecules could be a viable strategy to reverse key neuronal deficits.
2. Enhancing PARP1 Activity: The most direct and upstream intervention would be to promote DNA repair by stimulating PARP1 activity. A study shows that NAD supplementation in MECP2-null neurons can dramatically stimulate PARP1, reversing not only DNA damage and senescence but also restoring dendritic branching and metabolic function. This finding provides a rationale for exploring NAD supplementation as a potential clinical intervention.
3. Metabolic Support: The observation that MECP2-deficient cells can better utilize lipids for energy production is consistent with evidence of symptomatic relief in RTT patients on a ketogenic diet. This approach could provide a complementary therapeutic strategy to bypass the observed mitochondrial deficiencies.

## Genomic instability in neurons

In fact, there is a growing body of evidence supporting the idea that genomic instability is a hallmark of neurons and a major contributor to neurological disorders and aging (46–53). The brain has a high metabolic rate, consuming about 25% of the body’s glucose and oxygen. This intense metabolic activity generates a large number of reactive oxygen species (ROS) as a byproduct. ROS are highly reactive molecules that can directly damage DNA, leading to single- and double-strand breaks. Neurons clearly undergo dynamic and rapid changes in gene expression in response to neuronal activity. This process of transcription introduces a significant amount of topological stress on the DNA. To manage this, DNA must be constantly uncoiled and re-coiled, a process that relies heavily on enzymes like topoisomerases (54–58). The high rate of transcription in neurons means this process is a constant source of potential DNA damage.

Because neurons do not divide, they cannot use the robust DNA repair mechanisms associated with the cell cycle. As a result, DNA damage can accumulate in neurons over their lifetime. Studies have shown that neurons in the human brain can have a high number of copy number variations (CNVs), and that DNA damage such as double-strand breaks, are found to increase during physiological activities like learning and memory formation (54, 57). A link has been established between genomic instability and neurodegenerative diseases such as Alzheimer’s, Parkinson’s, and Huntington’s (48, 50, 52, 54, 59). Mutations in genes that encode DNA repair proteins or chromatin remodelers like the BAF complex are frequently associated with intellectual disabilities and other neurodevelopmental disorders, highlighting the critical importance of these pathways in maintaining neuronal health (60–62). Together the molecular hurdles faced by the unique functions of neurons perhaps make the stress caused by loss of MECP2 particularly acute in neurons.

## Metabolic dysfunction as a result of cellular stress

MECP2 deficiency appears to have consequences for cellular metabolism, particularly in relation to mitochondrial function (27, 63–65). Multiple lines of evidence indicate that MECP2-deficient cells exhibit severe metabolic dysfunction. Metabolic tracing experiments using C-13-Glucose reveal that MECP2-null neurons produced TCA cycle metabolites at a much lower rate compared to their wild-type counterparts, demonstrating diminished TCA cycle activity (64). Furthermore, a Seahorse assay, a measure of oxygen consumption and an indicator of TCA cycle activity, showed that MECP2-mutant neurons from different patients are defective at consuming oxygen and have a reduced ATP consumption rate. These findings are consistent with a study on mesenchymal stromal cells from a mouse RTT model, which found that MECP2 deficiencies led to impaired mitochondrial energy production showing that these cells retained metabolic flexibility, capable of switching between nutrients like glucose, palmitate, and glutamine to produce ATP (31). However, their basal respiration and ability to produce mitochondrial ATP in the presence of these fuels were significantly reduced, and their respiratory capacity was diminished when fueled by glucose or glutamine. This suggests a state of mitochondrial insufficiency rather than total failure. The observation that these cells could better cope with increased energy demand when fueled by palmitate is particularly notable, as it is consistent with reports of symptom relief in RTT patients on a ketogenic diet. Our own data suggest that these metabolic defects are a consequence of elevated DNA damage. Elevation of P53 in various contexts is known to lead to suppression of mitochondrial metabolism, and experiments with inducers of DNA damage in neurons clearly demonstrated this phenomenon.

Synaptic plasticity, a process crucial for learning and memory, is consistently reduced in RTT models (26, 66–68). A study on a senescence-accelerated mouse model showed that overexpression of MeCP2 could elevate synaptic plasticity and cognitive function, while its knockdown impaired these functions (26). This finding provides a direct functional link between MeCP2, synaptic health, and the cognitive decline characteristic of RTT. The evidence points to a clear causal relationship between the cellular-level pathologies and the macro-level neuronal deficits. The metabolic dysfunction and compromised mitochondrial activity are likely downstream consequences of the senescence program, which is activated by persistent DNA damage. The cell, sensing genomic stress, enters a state of senescence to halt proliferation and conserve energy. This state of compromised metabolic activity inhibits the maintenance and growth of complex dendritic arbors and the plasticity of synapses, leading to the functional deficits observed in RTT.

This model provides a compelling alternative to a purely transcriptional explanation for RTT. Rather than arguing that the symptoms are caused by the misexpression of a few key genes, it posits that the observed transcriptional changes (e.g., in synaptic and metabolic genes) are part of a coordinated cellular stress response. These changes are secondary to a more fundamental failure in genomic integrity and cellular maintenance. This view better explains why a consistent set of DEGs has been so difficult to identify across studies, as the transcriptional response may be highly contextual and cell-type-specific, while the underlying cause—DNA damage and senescence—remains conserved. The model also offers a novel perspective on RTT as a “premature aging” syndrome. The presence of pathologies like cellular senescence, DNA damage, and metabolic decline, which are all hallmarks of chronological aging, suggests that MECP2 plays a critical, conserved role in regulating cellular longevity. This new perspective potentially connects RTT to a broader range of age-related disorders.

On the other hand, Lasalle and others have proposed a model that share some features with ours including metabolism. In this case, the loss of MECP2 leads to hypoxia, which then triggers many of the same molecular and physiological phenotypes described here as triggered by DNA damage (69). Interestingly, this mechanism is also potentially related to PARP1 activity as HIF1 is known to be regulated by parylation. Future experiments should focus on determining whether PARP1 regulation by MECP2 as proposed in (44) leads to altered response to hypoxia as proposed by Lasalle (Table 1).

**Table 1:** Senescence and DNA damage phenotypes across RTT cell models.

Cell model	Key phenotypes observed	Source
Human Mesenchymal Stem Cells (MSCs)	Premature senescence, cell cycle arrest, reduced proliferation, elevated *β*-galactosidase, reduced apoptosis, DNA damage	(32)
Mouse Mesenchymal Stromal Cells (MSCs)	Premature senescence, cell cycle arrest (G1), reduced proliferation and apoptosis, impaired mitochondrial energy production	(31)
Human iPSC-derived Neurons	Induction of SASP, elevated β-galactosidase, p53/p21 induction, reduced dendritic branching, elevated *γ*-H2AX foci, increased comet tail moment, impaired DNA repair	(30, 44)
Human Brain (post-mortem)	DNA damage and senescence transcriptional signatures, p53 target gene induction	(43)
Rat Brain (Mecp2-null)	DNA damage and senescence transcriptional signatures, elevated γ-H2AX foci, increased comet tail moment	(43)
Murine Neuroblastoma Cells	Senescence, impaired neural differentiation, reduced proliferation, DNA damage	(27)

## Convergent mechanisms in chromatinopathies

The possibility that Rett could be a disorder of genomic stability highlights striking similarity with other chromatinopathies, suggesting that impaired DNA repair is a shared driver across this class of neurodevelopmental syndromes. Beyond Rett and Coffin-Siris syndrome, disorders such as Rubinstein-Taybi syndrome (caused by CBP/p300 mutations) and Cornelia de Lange syndrome (linked to Cohesin complex defects) display significant phenotypic overlap, including intellectual disability and microcephaly (70–75). Mechanistically, these conditions share a fundamental vulnerability: the proteins involved are essential not just for transcription, but for modulating chromatin architecture to allow access for repair machinery. Just as MECP2 deficiency prevents PARP1 from efficiently engaging damaged DNA, the loss of histone acetyltransferases in Rubinstein-Taybi or structural cohesins in Cornelia de Lange syndrome impedes the recruitment of Homology-Directed Repair (HDR) or Non-Homologous End Joining (NHEJ) factors. This parallel implies that the senescence-associated metabolic collapse observed in RTT is not unique, but rather a representative example of a broader stress response where the inability to maintain genomic integrity under the high metabolic load of the brain leads to a conserved pathway of neuronal dysfunction (Table 2).

**Table 2:** Key MECP2 interactors and their roles in DNA repair. Note that the data were pulled from BioGrid.

Interacting protein	Known function in DNA repair or chromatin	Source of finding (summarized in BioGrid)
PARP1	Mediator of DNA repair; adds poly-ADP-ribose (PAR) moieties to proteins.	IP-Mass Spec, Reciprocal Pulldown, External Databases
BAF complex components (SMARCA/B/C/D/E)	Opens chromatin to facilitate DNA repair; chromatin remodeling.	IP-Mass Spec, External Databases
XRCC5, DDB1	Components of DNA repair pathways.	IP-Mass Spec, External Databases
TOP1, TOP2B	Topoisomerase 1; involved in DNA replication and repair.	IP-Mass Spec, Reciprocal Pulldown, External Databases
NUCKS1	Promotes RAD54 activity in homologous recombination DNA repair.	IP-Mass Spec, External Databases

## Therapeutic implications

The framing of RTT as a disorder of genomic instability and cellular senescence unveils specific, actionable therapeutic targets beyond gene replacement and are summarized in Table 3. Given that chronic p53 activation drives the downstream deficit, but systemic p53 inhibition carries unacceptable oncogenic risks, targeting the senescent state itself offers a safer alternative. The application of senolytics, which selectively eliminate senescent cells, or senomorphics, which dampen the Senescence-Associated Secretory Phenotype (SASP), could mitigate the metabolic and synaptic dysfunction observed in RTT neurons. Furthermore, the discovery of the MECP2-PARP1 interaction suggests that restoring DNA repair capacity is a critical upstream intervention. Since PARP1 activity is suppressed in MECP2-deficient cells, therapeutic strategies that stimulate PARP1—either through direct small-molecule activators or by supplementing with NAD + precursors to fuel PARP1 enzymatic activity—could ameliorate the accumulation of DNA breaks. These approaches represent a dual-pronged strategy: enhancing the neuron’s intrinsic capacity for genomic maintenance via PARP1 while simultaneously alleviating the physiological stress of premature senescence.

**Table 3:** Functional deficits and potential therapeutic interventions.

Observed RTT defect	Experimental intervention	Outcome of intervention	Source
Dendritic branching defects	P53 inhibition (Pifithrin-α)	Restored dendritic complexity	(30)
DNA damage, senescence, dendritic branching, metabolic dysfunction	PARP1 stimulation (NAD supplementation)	Reversed DNA damage, senescence; restored branching and metabolism	(44)
Impaired mitochondrial function	N/A	Symptomatic relief from ketogenic diet suggests metabolic support is beneficial.	(28, 64, 76–78)
Synaptic plasticity deficits	MeCP2 overexpression	Elevated synaptic plasticity and cognitive function	(66-68, 79-81)

## Future questions

The findings have broader implications for a class of neurodevelopmental disorders. The presence of similar DNA damage and senescence phenotypes in CDKL5 deficiency disorder suggests a shared pathological etiology, which could lead to common therapeutic strategies for multiple diseases. However, several key questions remain. Future research should aim to elucidate the precise molecular mechanism of the MECP2-PARP1 interaction. Is MECP2 a scaffold protein that recruits PARP1 to sites of DNA damage, or does it modulate PARP1’s activity through other means? These findings, while robust in human and rat models, need to be validated in RTT organoids and transgenic animal models to confirm their clinical relevance and test the efficacy of therapeutic interventions in a pre-clinical setting.

## Conclusion

The synthesis of the provided research fundamentally redefines our understanding of Rett Syndrome, shifting the central narrative from a disease of transcriptional misregulation to a pathology rooted in a failure of genomic integrity and cellular homeostasis. The evidence points to a new model, where MECP2 deficiency directly impairs DNA repair, leading to a state of chronic genotoxic stress. This stress triggers a senescence program that ultimately drives the hallmark metabolic and neuronal deficits of the disease. This shift not only offers a deeper mechanistic understanding of RTT but also provides a new, actionable framework for developing targeted therapeutic strategies. The potential to reverse key disease phenotypes by modulating pathways like DNA repair and senescence offers hope for patients and provides a valuable lens for understanding a broader range of age-related neurodegenerative disorders.
